# Primary cardiac lymphoma treated with R-CHOP resulting in rhythm recovery and avoidance of permanent pacing: a case report

**DOI:** 10.1093/ehjcr/ytag185

**Published:** 2026-03-10

**Authors:** Robert Ambrogetti, Rajaperumal Ravi, Eaint Kay Khine Thein, Sina Fathieh, Rodney De Palma

**Affiliations:** Department of Cardiology, Wycombe Hospital, Buckinghamshire Healthcare NHS Trust, Queen Alexandra Rd, High Wycombe HP11 2TT, UK; Department of Cardiology, Wycombe Hospital, Buckinghamshire Healthcare NHS Trust, Queen Alexandra Rd, High Wycombe HP11 2TT, UK; Department of Cardiology, Wycombe Hospital, Buckinghamshire Healthcare NHS Trust, Queen Alexandra Rd, High Wycombe HP11 2TT, UK; Department of Cardiology, Wycombe Hospital, Buckinghamshire Healthcare NHS Trust, Queen Alexandra Rd, High Wycombe HP11 2TT, UK; Department of Cardiology, Wycombe Hospital, Buckinghamshire Healthcare NHS Trust, Queen Alexandra Rd, High Wycombe HP11 2TT, UK

**Keywords:** Lymphoma, Primary cardiac lymphoma, Cardiac mass, Syncope, Arrhythmia, Case report

## Abstract

**Background:**

Primary cardiac lymphoma (PCL) is a rare, aggressive extranodal lymphoma involving the heart and pericardium. It often presents with nonspecific symptoms and conduction disturbance, resulting in diagnostic delay. Multimodal cardiac imaging can characterize PCL tumours and guide biopsy, while histology is required for definitive diagnosis and treatment.

**Case Summary:**

A man in his 60s with well-controlled HIV presented with recurrent syncope preceded by fever, chest pain, and fatigue. He was found to have profound first-degree atrioventricular block (PR interval 414 ms). Transthoracic echocardiography revealed a large interatrial septal mass protruding into both atria with close association to the aortic root. Cardiac CT, MRI, and FDG PET–CT confirmed a metabolically active mass extending around the peri-aortic structures. Transoesopheal-guided biopsy confirmed PCL of B-cell origin. R-CHOP chemotherapy was commenced with continuous cardiac monitoring. Despite paroxysmal atrial flutter and intermittent higher-grade AV conduction disturbance, permanent pacing was deferred. After two cycles of rituximab, cyclophosphamide, doxorubicin, vincristine, and prednisolone (R-CHOP), imaging showed substantial tumour regression, reduced FDG avidity, and improved AV conduction (PR 250 ms) and resolution of syncope.

**Discussion:**

PCL can present with cardiogenic syncope due to tumour-related involvement of the conduction system and mass effect. This case highlights the value of early multimodal imaging to rapidly characterize cardiac masses and monitor treatment response, while emphasizing the necessity of tissue diagnosis. In selected stable patients, early chemotherapy may reverse tumour-related conduction disease, allowing for deferral of permanent pacemaker implantation.

Learning points

1. High-grade AV block/bradyarrhythmia from PCL may reverse with chemotherapy. Hence, selected clinically stable patients may not need permanent pacing.
2. Early multimodality imaging (echo, CT, MRI, PET–CT) is key for detecting, characterizing, and monitoring cardiac tumours.
3. Multidisciplinary care across cardiology and haematology are key to improving outcomes in cardiac lymphoma.

## Introduction

Primary cardiac lymphoma (PCL) is a rare extranodal non-Hodgkin lymphoma (NHL) that involves the heart and/or pericardium and can present with nonspecific symptoms, often resulting in diagnostic delay.^1^ Conduction abnormalities and arrhythmia may occur when tumour infiltration or pericardial extension involves the cardiac conduction system, with syncope as a potential presenting manifestation.^1^ Multimodal cardiac imaging can rapidly characterize PCL tumours, define anatomical relationships to critical structures, and support treatment-response assessment. However, histological diagnosis is required to confirm PCL and guide chemotherapy and prognosis.^2^

This case reports PCL presenting with syncope and atrioventricular conduction delay, where early biopsy enabled prompt treatment with rituximab, cyclophosphamide, doxorubicin, vincristine, and prednisolone (R-CHOP) and close rhythm surveillance. It highlights that, in selected stable patients with PCL, tumour regression with chemotherapy may reverse tumour-related conduction disease and allow deferral of permanent pacemaker implantation.

## Summary figure

**Figure:**
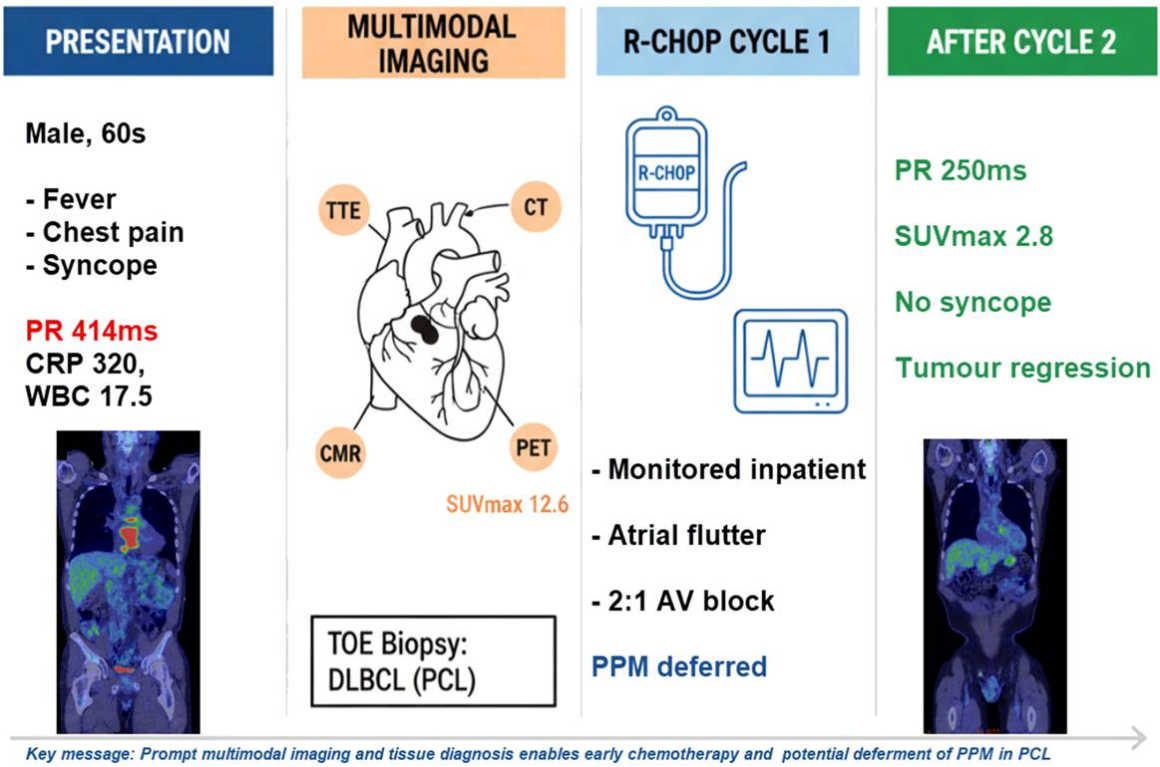


## Case presentation

A man in his 60s with a past medical history of well-controlled HIV and hypertension presented to his local hospital after two syncopal episodes. Both episodes were followed by spontaneous and complete recovery with no postevent confusion. These syncopal episodes were preceded by 2 days of fever, central chest pain, and progressive fatigue.

His vital signs, including blood pressure, temperature, and peripheral oxygen saturation, were within normal limits. His cardiovascular and neurological physical examinations were unremarkable.

Initial blood tests showed elevated inflammatory markers (CRP 320 mg/L, WBC 17.5 109/L), negative serial troponins (Troponin I 20.9 ng/L and 26.6 ng/L), positive IgG for EBV and CMV, undetectable HIV viral load, and a mild reduction in CD4 count (see Supplementary material online, *Table S1*).

**Figure 1:**
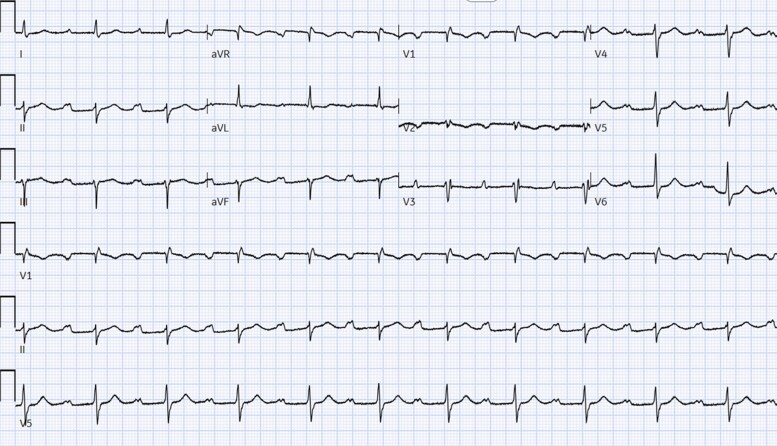
12-lead ECG showing a PR interval of 414 ms and a partial right bundle branch block with left axis deviation (left anterior fascicular block).

ECG on admission showed a marked first-degree heart block (PR interval 414 ms), an incomplete right bundle branch block, and left axis deviation (left anterior fascicular block) (*Figure 1*).

Transthoracic echocardiography was performed, which revealed a large echo-reflective structure in the atrial septal wall protruding into both the left and right atria (*Figure 2.1* and Supplementary material online, *Video S1*). Other than a small, simple global pericardial effusion, there were no other significant echocardiographic findings.

**Figure 2:**
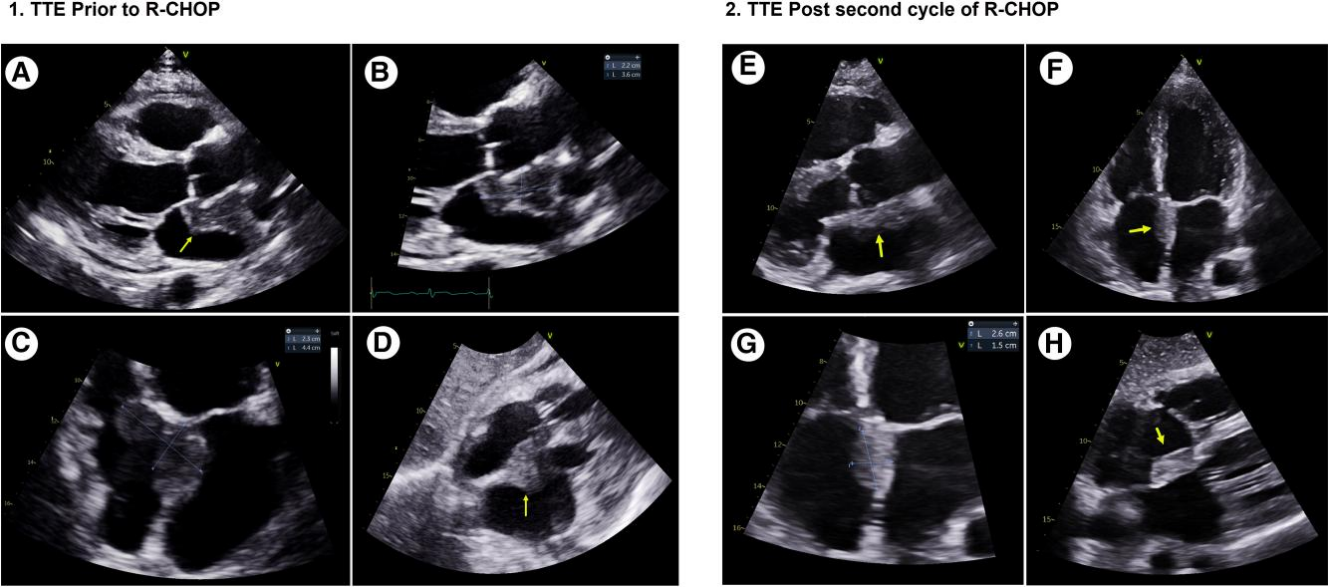
1.TTE images pre R-CHOP. (1*A*) Parasternal long axis (PLAX) showing the atrial mass encircling the aortic root (yellow arrow). (1*B*) Focused PLAX showing the atrial mass 2.2 × 3.6 cm. (1*C*) Apical four chamber(A4CH) zoomed showing intra atrial septal mass, 2.3 × 4.4 cm. (1*D*) Subcostal view showing the intra-atrial mass (yellow arrow). (2) TTE images post second R-CHOP cycle. (2*E*) Zoomed PLAX showing the tumours close association with the aortic root (yellow arrow). (2*F*) A4CH showing the intra-atrial septal location of the tumour (yellow arrow). (2*G*) Zoomed A4CH showing the atrial tumour size, 2.6 × 1.5 cm. (2*H*) Subcostal view showing the intra-atrial septal tumour with protrusion into the right atrium (yellow arrow).

Cardiac malignancies, including both primary benign and malignant, and secondary metastasis to the heart, were considered. Given the patient’s background of HIV, although uncommon, lymphoma was considered early on. A peri-aortic abscess was considered early based on syncope with marked PR prolongation, fever, elevated inflammatory markers, background of HIV, and the lesions proximity to the aortic root. In view of minimal aortic regurgitation, this became less likely, but empirical antibiotics were started, and cardiothoracic input was sought.

**Figure 3:**
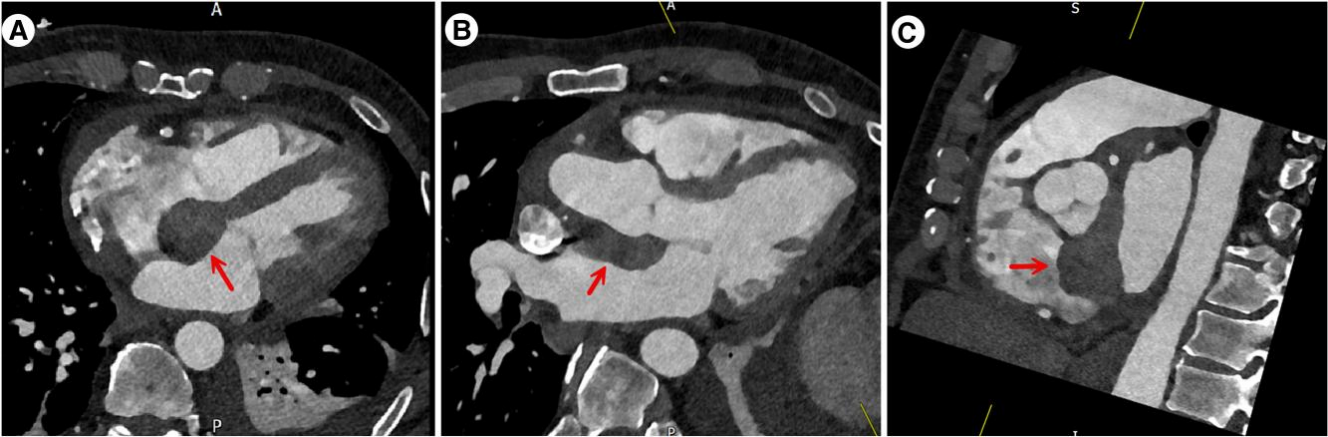
Cardiac CT scan. (*A*) Four chamber diastolic view, the red arrow highlights the mass in the atrial septum protruding into the left and right atria. (*B*) Modified axial slice showing the left ventricular outflow tract and aorta. The red arrow highlights the septal mass position in relation to the aortic root *C*. Modified short-axis view across the base of the heart shows the atrial septal mass (red arrow) association with the noncoronary and right coronary cusps.

A cardiac CT (CTCA) was performed, which revealed a soft tissue dense mass up to 2.7 cm attached to the interatrial septum. The mass extended to the aortic root adjacent to non and right coronary cusps, encasing the left coronary artery from the left main stem to proximal left anterior descending (4 cm in total length). Mixed plaques were also noted at the LAD and RCA, causing mild luminal stenosis (*Figure 3*).

Cardiac MRI (CMR) showed an irregular mediastinal mass arising in the pericardium, extending to the atrial septal space, and enveloping the aorta from the aortic root to the aortic arch. Maximum measurement of 9 cm in cranio-caudal direction and ∼2 cm in AP direction. There was no myocardial involvement. With elevated T1 mapping values (∼1300 ms) and T2 mapping values (76–84 ms), hyperintensity on T2-weighted STIR images, and enhancement on first-pass perfusion sequences, the CMR findings were consistent with an active malignant solid tissue mass arising from the pericardium (see Supplementary material online, Figure  *S1*).

A PET 18F-FDG (CT–PET) whole body scan showed marked avidity of the intra-atrial mass. Avidity extended caudally and medially to the inferior vena cava and cranially to the posterior right main pulmonary artery, with a maximum standardized uptake value (SUV_max_) of 12.6 (*Figure 4.1*).

**Figure 4:**
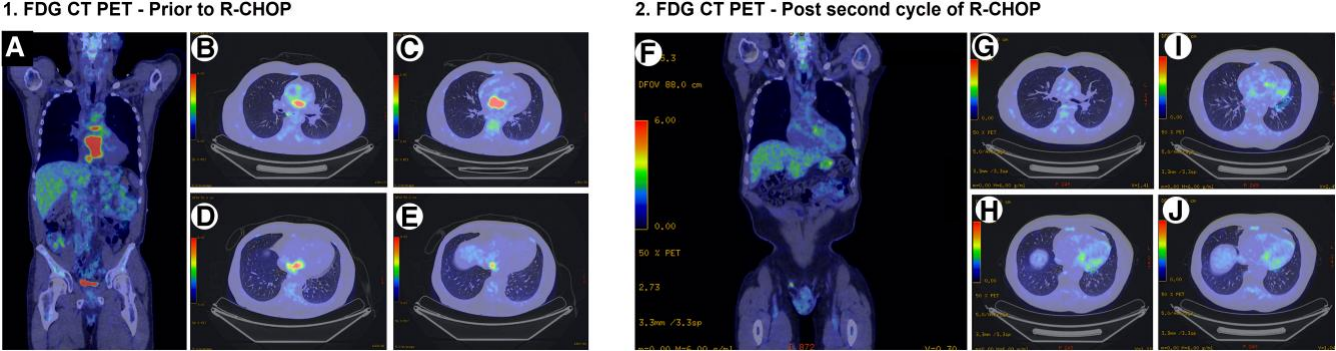
(1) CT PET scan showing marked uptake in the heart. (1*A*) Coronal whole body FDG PET showing areas of avidity highlighted in red. 1*B–E*. Areas of avidity are shown in red extending from the IVC through the intra-atrial septum cranially. (2) CT PET showing reduced avidity. (2*F*) Coronal slice showing reduced avidity compared to the pretreatment scan. (2*G–J*) Axial slices from the precarinal level to the IVC showing marked reduction in avidity throughout the heart and surrounding structures.

Prevascular and subcarinal nodes were mildly avid (SUV_max_ 3.2). There was no cervical, supraclavicular, or subdiaphragmatic lymphadenopathy. An incidental right testicular lesion did not show any increase in avidity.

For diagnostic confirmation, a transoesophageal echocardiogram-guided biopsy was performed. Samples were sent for histopathological analysis, which showed atypical large B-cells immunopositive for LCA, CD20, CD79a, CD30, and BCL6, confirming the diagnosis of PCL, specifically a diffuse large B-cell lymphoma.

**Figure 5:**
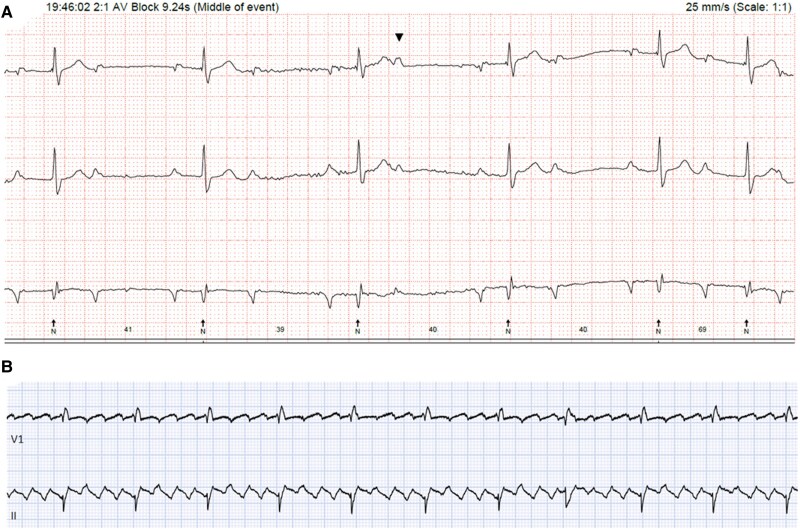
(*A*) Holter monitor tracing showing 2:1 heart block. (*B*) Rhythm strip trace showing atrial flutter.

Following diagnosis of PCL, the patient was initiated on R-CHOP chemotherapy under the guidance of haematology. Given his prolonged PR interval at baseline, chemotherapy was commenced under continuous cardiac monitoring in the cardiology ward. On the first night of treatment, cardiac monitoring showed runs of atrial flutter, a single episode of 2:1 heart block, and Wenckebach (*Figure 5A and B*).

A multidisciplinary team meeting decision (MDT) was made not to implant a permanent pacemaker at this stage. This decision was based on the patient being asymptomatic and the potential for tumour regression with the initiation of treatment. He was started on anticoagulation (CHA_2_DS_2_-VASc 1) for atrial flutter.

The right testicular tumour was diagnosed as a seminoma and was surgically resected. No further treatment for this incidental finding was necessary.

The patient was discharged to his usual residence after completing the first of six cycles R-CHOP chemotherapy. After his second cycle of treatment, echocardiography demonstrated a significant reduction in tumour size from 2.3 × 4.4 to 2.6 × 1.5 cm (*Figure 2.2*, Supplementary material online, *Video S2*).

**Figure 6:**
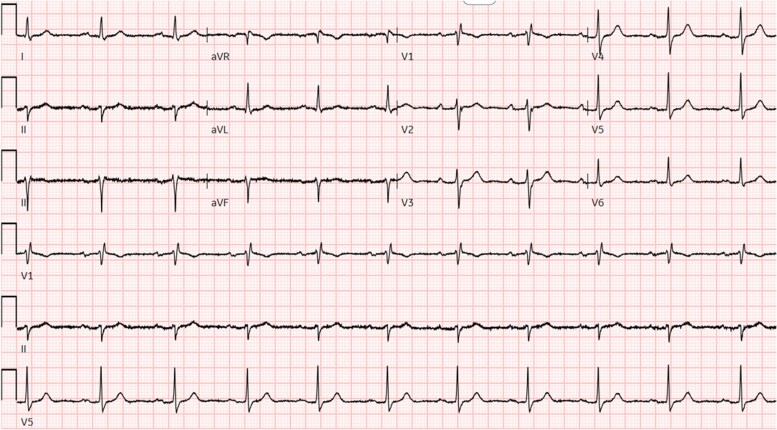
ECG post second cycle of chemotherapy shows PR interval of 250 ms. Right bundle branch block and left anterior fascicular block persist.

A follow-up 12-lead electrocardiogram showed sinus rhythm with first-degree atrioventricular (AV) block and a PR interval of 250 ms. While the PR interval shortened, the incomplete right bundle and left anterior fascicular block persisted (*Figure 6*).

There has been no further documented bradyarrhythmia, and the patient reported no further pre–syncopal or syncopal episodes.

A restaging PET CT after his second cycle of R-CHOP treatment showed a significant reduction in avidity compared to the pretreatment scan (SUV_max_ 2.8) (*Figure 5*). Prevascular lymph nodes no longer show any avidity (Deauville 2–3).

Ongoing monitoring will include serial ECGs and cardiac imaging with each chemotherapeutic cycle. After completion of chemotherapy, he will remain under routine guideline-directed cardiac and haematological follow-up.

## Discussion

PCL is a rare type of non–Hodgkin lymphoma, defined as an extranodal lymphoma involving only the heart and/or pericardium.^3^ Primary malignant cardiac tumours account for approximately 34 cases per 100 million persons.^4^ PCL accounts for 1–2% of primary cardiac tumours and 0.5% of extranodal lymphomas.^3^ Up to 90% of PCL cases are diffuse B-cell NHL.^3^ PCL is more common in males (∼2:1 male-to-female ratio), Caucasians (∼80%), and immunocompromised patients.^3^ Cases have been reported in children and older adults. However, PCL tends to be a haematological malignancy of the older adult with a median age at diagnosis of 63 years.^3^

PCL often presents with nonspecific symptoms, including chest tightness, palpitations, dyspnoea, constitutional symptoms, and syncope. The most common cardiac manifestations are brady and tachyarrhythmias, pericardial effusion, and heart failure.^3,5^ The location of the PCL in the heart and its relationship to surrounding structures influence the presenting symptoms. The right atrium (86 −92%) is the most common site for PCL tumours to manifest, and this can sometimes result in unusual presentations, including thromboembolism and superior vena cava syndrome.^1,3,5^ A predilection to right-sided involvement in PCL is thought to be due to lymphatic drainage into the superior vena cava, exposing the right heart to antecedent occult systemic nodal lymphoma.^3^ PCL tumours have also been reported to arise in the left ventricle and left atrium and to have variable valvular, myocardial, and pericardial involvement.^3,5^ Intra-atrial septal involvement has been reported to occur in up to 41% of cases.^3^ Tumour extension along epicardial surfaces encasing coronary arteries and the aorta is considered a typical radiological finding in PCL.^6^ In the above case, an intra-atrial septal location and extension of the PCL to the aortic root likely explain the prolonged PR interval and conduction disturbances that led to his presentation with syncope. Patients with PCL who present due to arrhythmia have been noted to have a better prognosis than those with no rhythm disturbances.^3^ This is likely due to earlier presentations and the detection of arrhythmia, resulting in further investigations such as cardiac imaging, as seen in our case.^3^ Cases of PCL, such as ours, where bradyarrhythmia has regressed with treatment without requiring a pacemaker, have previously been reported.^3,7^ His chest pain was likely explained by pericardial involvement of PCL and encasement of LMS and LAD coronary arteries.

The differential diagnosis for atrial masses includes thrombus, infectious masses (e.g. intracardiac abscesses), and malignancy (both primary and secondary).^8^ In this case, thrombus was considered less likely given the absence of typical risk factors and its broad-based and immobile appearance involving both sides of the interatrial septum on TTE.^8^ Infective peri-aortic process was considered early on due to the background of HIV, fever, raised inflammatory markers, proximity to the aorta, and prolonged PR interval. However, minimal aortic regurgitation and valvular involvement made this less likely.^8^ Given the lesion’s location on the atrial septum, lipomatous hypertrophy and myxoma, histologically benign lesions, were other possible differentials. However, the morphological appearances on TTE are not typical of either.^8^ Historically, PCL diagnosis was most frequently made at autopsy postmortem. However, as seen in our case, modern technology and advances in multimodal cardiac imaging permit early detection, diagnosis, and treatment.^3^ TTE is the first-line imaging investigation. TTE can provide information on location and morphology and evaluate haemodynamic impact.^8^ TTE has a reported sensitivity of 60% for demonstrating tumours in PCL.^1,2^ Hence, further imaging is often required in the form of a combination of CT, CMR, and PET-CT. As seen in our case, TTE, CT, CMR, and PET–CT imaging modalities add clinically pertinent information incrementally and have crucial roles in the detection and characterization of cardiac masses. However, in PCL, biopsy and histological diagnosis with immunohistochemical profiling is required for definitive diagnosis to guide treatment and prognosis.^1^ PET–CT is particularly useful for monitoring treatment response in PCL.^6,8^ Our patient has had a significant reduction in tumour activity after two cycles of R-CHOP treatment. Extracardiac disease, advanced stage, left ventricular, and myocardial involvement are all associated with significantly worse outcomes.^3^ Hence, early imaging, especially CMR and FDG PET, can provide important prognostic information.

Due to its aggressive nature, PCL has historically been associated with poor prognosis. If left untreated, median survival has been reported as low as 1 week to several months. In modern times, early diagnosis through cardiac imaging and subsequent treatment has resulted in a significant improvement in prognosis, in some cases, curative.^1,5^ An analysis of 184 patients with PCL from Yin *et al.*^9^found that chemotherapy was the only treatment that improved survival. Isolated surgical intervention has not been shown to provide a mortality benefit in PCL.^1,9^ Hence, surgical intervention is often limited to a palliative measure or cases with obstructive symptoms necessitating emergency intervention.^9^ Chemotherapy regimens are guided by immunohistochemical profiling and are similar to those used in other types of NHL, such as CHOP or R-CHOP.^1^ Contemporary treatments, such as chimeric antigen receptor T-cell and bispecific antibody therapies, have been used in relapsed/refractory aggressive large B-cell lymphoma.^10^ With curative potential and the possibility for unique complications, further research is needed to understand their role in the treatment of PCL.

## Conclusion

PCL is a rare condition that, without prompt treatment, carries a fatal prognosis. The nonspecific and wide variety of possible clinical manifestations poses a challenge for clinicians to make an early diagnosis. As highlighted in this case, the prompt progression to definitive diagnosis via biopsy and histological analysis should be guided by early multimodal imaging. In those presenting with symptomatic bradycardia, prompt treatment can reverse conduction abnormalities and obviate the need for permanent pacing. Awareness of this rare but treatable condition is essential for timely diagnosis and favourable outcomes.

## Supplementary Material

ytag185_Supplementary_Data

## Data Availability

All relevant data, including de-identified imaging and laboratory results, are provided within the article and or supplementary documents; no additional datasets were used or generated.

## Lead author biography

Robert Ambrogetti is currently a Speciality trainee in Cardiology and General Internal Medicine at Buckinghamshire Health Care Trust, UK. He is a cardiology specialty trainee at Wycombe Hospital Buckinghamshire Healthcare Trust, UK. His clinical interests include interventional cardiology, cardiac imaging, sports cardiology, general cardiology, and medicine.

## Notes

**Consent:** The authors confirm that written informed consent for publication of the case details and associated images was obtained from the patient in accordance with COPE guidelines and the European Heart Journal—Case Reports editorial policies
