# Supplementary material for: Primary cardiac lymphoma treated with R-CHOP resulting in rhythm recovery and avoidance of permanent pacing: a case report
Source: Eur Heart J Case Rep. 2026 Mar 10;10(4):ytag185. doi: 10.1093/ehjcr/ytag185 (PMC13042225; doi:10.1093/ehjcr/ytag185)
Supplement: ytag185_Supplementary_Data [file ytag185_supplementary_data.zip › Supplements.docx]

Supplements

Table 1.

| **Investigation** | **Result** | **Units** | **Normal range** |
| --- | --- | --- | --- |
| HB | 126 | g/L | 130 - 180 |
| WBC | 17.5 | 10^9^/L | 3.7 - 11 |
| CRP | 320 | mg/L | 0-5 |
| ESR | 34 | mm/hr |  |
| Procalcitonin | 0.1 | ng/ml |  |
| CR | 70 | umol/L | 63-111 |
| CK | 123 | U/L |  |
| Troponin | 26.6 | ng/L |  |
| NT-Pro BNP | 339 | ng/L |  |
| TSH | 1.49 | mIU/L | 0.35-4.95 |
| PSA | 0.21 | ng/ml | 0-4.5 |
| CA19-9 | <2.0 | U/mL | 0-37 |
| CEA | 1.1 | ug/L | 0-5 |
| Lactate | 1 | mmol/L |  |
| Total lymphocyte count | 0.49 | 109/L | 1.0 - 2.8 |
| Total T cells | 0.32 | 109/L | 0.7 - 2.1 |
| Total T cells % | 66.3 | % | 55 - 83 |
| Total CD4+ T cells | 0.11 | 109/L | 0.3 - 1.14 |
| CD4% to total lymphocytes | 21.8 | % | 28 - 57 |
| Total CD8+ T cells | 0.2 | 109/: | 0.2 - 0.9 |
| CD8% of total lymphocytes | 41.5 | % | 10 --39 |
| CMV IgM | not detected |  |  |
| CMV IgG | detected |  |  |
| EBV IgG | detected |  |  |
| HIV RNA (PCR) | not detected |  |  |
| HIV VL log (10) copies /ml | not detected |  |  |

Supplementary Fig 1


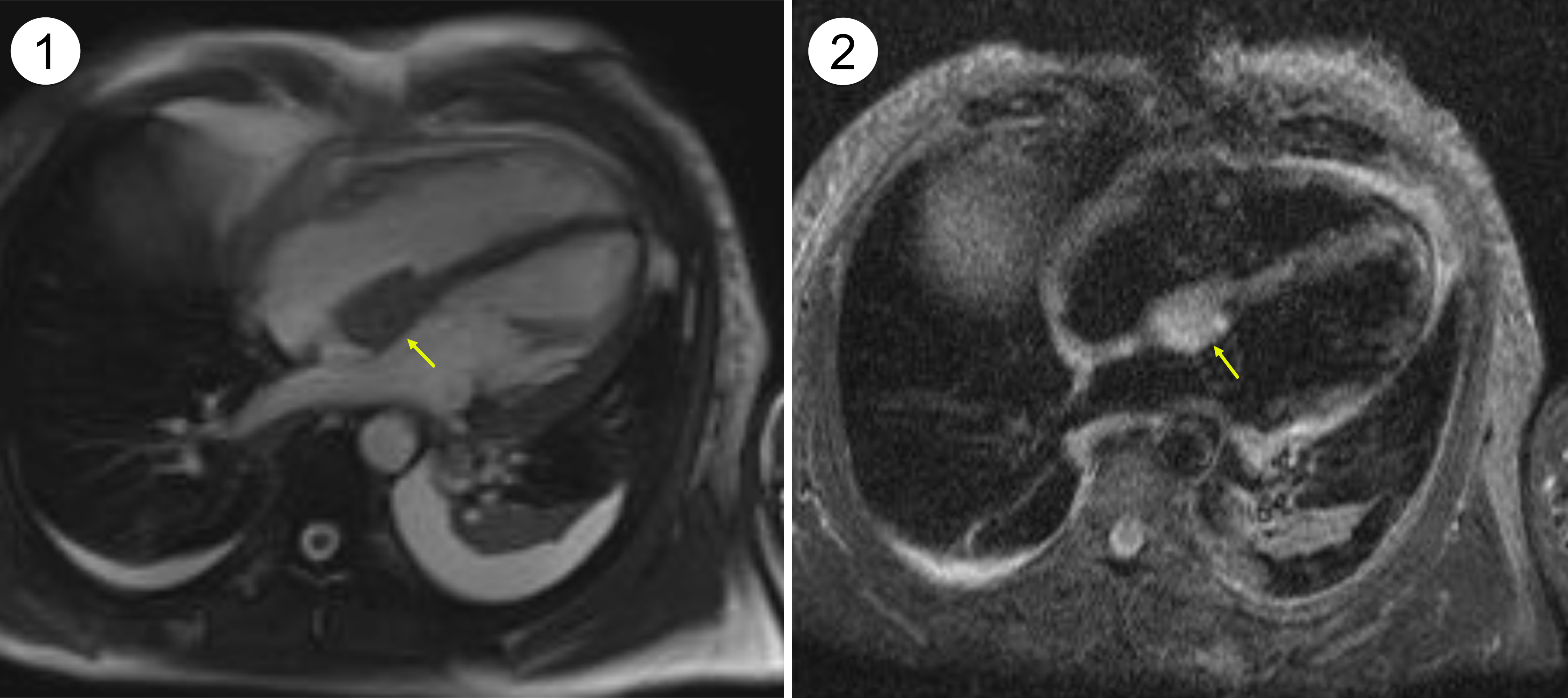


**Figure 4:** Cardiac MRI 1. Horizontal long axis time of flight image showing intra – atrial mass marked by yellow arrow. 2. T2 stir images showing hyperintensity of the intra-atrial mass marked at the yellow arrow.
